# Prescription Drug Costs Among People With Alzheimer Disease and Related Dementias

**DOI:** 10.1001/jamanetworkopen.2024.33026

**Published:** 2024-09-12

**Authors:** Seyeon Jang, Rozalina G. McCoy, Jie Chen

**Affiliations:** 1 Department of Health Policy and Management, School of Public Health, University of Maryland, College Park; 2 University of Maryland Center on Aging, College Park; 3 The Hospital and Public Health Interdisciplinary Research Lab, School of Public Health, University of Maryland, College Park; 4 University of Maryland Institute for Health Computing, Bethesda; 5 Division of Endocrinology, Diabetes, and Nutrition, Department of Medicine, University of Maryland School of Medicine, Baltimore

## Abstract

This cross-sectional study examines the association of Alzheimer disease and related dementias with prescription drug expenditures across cost distributions.

## Introduction

Prescription drug expenditures increase costs for the care of patients with Alzheimer disease and related dementias (ADRD), posing a substantial burden on patients, their families, and Medicare.^1,2^ Although several studies^1,2,3^ have examined average drug expenditures by individuals with ADRD, these costs exhibited skewed distributions, indicating substantial variations in financial burden.^3^ The lower end of drug cost distribution may suggest inequitable access, whereas the higher end may indicate more severe or complex health needs.^3^ Applying quantile regression analysis,^3^ we examined the associations between ADRD and prescription drug expenditures across cost distributions.

## Methods

This cross-sectional study used the 2003 to 2021 Medical Expenditure Panel Survey (MEPS), a nationally representative dataset among community-dwelling individuals in the US,^4^ to calculate the annual total and out-of-pocket (OOP) prescription drug expenditures per person, adjusted to 2022 US dollars.^5^ We report the mean and 25th, 50th, 75th, and 90th percentiles of total and OOP drug costs for individuals with and without ADRD. The subpopulation analysis restricted to individuals with ADRD examined the association of coexisting chronic conditions with drug costs.

Sampling weights were applied to account for survey nonresponses, and results are nationally representative.^4^ Analyses were performed using Stata statistical software version 16 (StataCorp) between March and April 2024. Our study followed the STROBE reporting guidelines and was exempt from institutional review board review and informed consent owing to the use of publicly available, deidentified data, in accordance with 45 CFR §46.

## Results

We identified 77 911 community-dwelling adults aged 65 years and older without ADRD (43 147 women [55.4%]; mean [SD] age, 73.8 [6.4] years) and 3046 adults (3.8%) with ADRD (1989 women [65.3%]; mean [SD] age, 80.8 [5.5] years). Individuals with ADRD incurred annual mean total drug expenditures of $4465.3 (95% CI, $4150.7-$4780.0) and OOP costs of $1199.7 (95% CI, $1102.0-$1297.4) (Table 1). At the 90th percentile of drug cost distributions, people with ADRD experienced $10 054.1 (95% CI, $9387.7-$10 720.4) total and $2952.2 (95% CI, $2645.4-$3259.0) OOP drug costs, compared with $6746.1 (95% CI, $6632.6-$6859.6) total and $1697.9 (95% CI, $1666.3-$1729.5) OOP costs for those without ADRD.

**Table 1.** Comparison of Weighted, Unadjusted Prescription Drug Expenditures Between Groups With and Without ADRD^a^

Prescription expenditures	No ADRD (n = 77 911)		Any ADRD (n = 3046)	
	Cost, mean (95% CI), $	*P* value	Cost, mean (95% CI), $	*P* value
Total				
Any total prescription, No. (%), individuals	70 442 (90.4)	NA	2918 (95.8)	NA
Total prescription if any	3137.6 (3043.6-3231.6)	<.001	4465.3 (4150.7-4780.0)	<.001
Total Q1 (25%)	228.6 (221.8-235.4)	<.001	754.3 (648.7-859.9)	<.001
Total Q2 (50%)	1043.4 (1022.1-1064.7)	<.001	2474.3 (2291.6-2657.1)	<.001
Total Q3 (75%)	3152.9 (3101.4-3204.3)	<.001	5686.3 (5316.6-6056.0)	<.001
Total Q4 (90%)	6746.1 (6632.6-6859.6)	<.001	10 054.1 (9387.7-10 720.4)	<.001
OOP				
Any OOP prescription, No. (%), individuals	67 497 (86.6)	NA	2828 (92.8)	NA
OOP prescription if any	772.5 (753.2-791.7)	<.001	1199.7 (1102.0-1297.4)	<.001
OOP Q1 (25%)	51.4 (49.6-53.2)	<.001	118.4 (101.1-135.7)	<.001
OOP Q2 (50%)	239.7 (235.0-244.4)	<.001	447.3 (401.3-493.3)	<.001
OOP Q3 (75%)	738.6 (725.6-751.6)	<.001	1301.3 (1189.3-1413.3)	<.001
OOP Q4 (90%)	1697.9 (1666.3-1729.5)	<.001	2952.2 (2645.4-3259.0)	<.001

Abbreviations: ADRD, Alzheimer disease and related dementias; NA, not applicable; OOP, out-of-pocket; Q, quantile.

^a^ Values represent the unadjusted amount of money spent on prescription drugs across the 25th to 90th percentiles of the drug cost distributions with 95% CIs. The sample included older adults aged 65 years and older with and without ADRD from the 2003 to 2021 Medical Expenditure Panel Survey data. ADRD diagnosis was identified by *International Classification of Diseases, Ninth Revision* diagnostic codes 290, 294, 331, or 797 for 2003 to 2015 data and by *International Statistical Classification of Diseases and Related Health Problems, Tenth Revision *diagnosis codes F01, F03, G30, and G31 for 2016 to 2021 data. The quantile results include zero expenditure.

The incremental total drug expenditures attributable to ADRD ranged from $410.4 (95% CI, $309.0-$511.8) at the 25th quantile (Q1) to $3683.8 (95% CI, $3013.8-$4353.7) at the 90th quantile (Q4) of total drug costs, whereas incremental OOP expenditures ranged from $46.5 (95% CI, $29.5-$63.5) at Q1 to $642.9 (95% CI, $416.1-$869.7) at Q4 (Table 2). Among individuals with ADRD, diabetes incurred the highest incremental total drug expenditures, which ranged from $1185.5 (95% CI, $863.8-$1507.1) in Q1 to $5217.5 (95% CI, $3970.9-$6464.0) in Q4. Importantly, all comorbidities incurred substantial costs, with individuals in Q4 experiencing additional drug expenditures of $1933.3 (95% CI, $1111.4-$2755.3) for depression, $1524.2 (95% CI, $607.1-$2441.3) for hypertension, and $2484.8 (95% CI, $1631.2-$3338.4) for heart disease. Similar patterns were observed for OOP costs (Table 2).

**Table 2.** Weighted Quantile Regression Results of Total and OOP Prescription Drug Costs Among Total Sample and People With ADRD^a^

Variable	Quantile 1		Quantile 2		Quantile 3		Quantile 4	
	Cost, mean (95% CI), $	*P* value	Cost, mean (95% CI), $	*P* value	Cost, mean (95% CI), $	*P* value	Cost, mean (95% CI), $	*P* value
Total sample (N = 79 998)								
Total prescription costs	410.4 (309.0-511.8)	<.001	1132.6 (971.7-1293.5)	<.001	2263.2 (1907.4-2619.0)	<.001	3683.8 (3013.8-4353.7)	<.001
OOP prescription costs	46.5 (29.5-63.5)	<.001	135.3 (101.6-169.0)	<.001	404.7 (326.3-483.1)	<.001	642.9 (416.1-869.7)	<.001
People with ADRD (n = 2877)								
Total prescription costs								
Diabetes	1185.5 (863.8-1507.1)	<.001	2228.8 (1694.5-2763.1)	<.001	3996.7 (3151.3-4842.0)	<.001	5217.5 (3970.9-6464.0)	<.001
Depression	757.8 (605.6-909.9)	<.001	1195.6 (832.9-1558.4)	<.001	1718.0 (1318.3-2117.8)	<.001	1933.3 (1111.4-2755.3)	<.001
Hypertension	210.0 (104.6-315.4)	<.001	376.4 (131.1-621.8)	.003	544.8 (170.2-919.5)	.004	1524.2 (607.1-2441.3)	.001
Heart disease	519.8 (411.0-628.7)	<.001	767.3 (512.9-1021.7)	<.001	1419.3 (1003.3-1835.4)	<.001	2484.8 (1631.2-3338.4)	<.001
OOP prescription costs								
Diabetes	72.4 (43.8-101.0)	<.001	181.3 (93.6-269.0)	<.001	246.6 (80.3-413.0)	.004	843.0 (331.2-1354.8)	.001
Depression	116.4 (77.3-155.6)	<.001	237.4 (178.0-296.8)	<.001	339.6 (222.3-456.9)	<.001	262.1 (−47.1-571.2)	.10
Hypertension	48.4 (22.5-74.4)	<.001	95.5 (42.4-148.5)	<.001	87.4 (−16.0-190.7)	.10	34.9 (−305.5-375.3)	.84
Heart disease	71.3 (45.3-97.3)	<.001	96.1 (45.7-146.5)	<.001	143.9 (42.4-245.4)	.005	320.5 (33.0-608.0)	.03

Abbreviations: ADRD, Alzheimer disease and related dementias; OOP, out-of-pocket.

^a^ Data represent the annual incremental prescription drug expenditures compared with their references (ie, not having the specific disease) of the total sample population or individuals diagnosed with ADRD across the 25th, 50th, 75th, and 90th percentiles (ie, quantiles) of total and OOP cost distributions. Age, age squared, gender, race and ethnicity (non-Hispanic White, non-Hispanic Black, Hispanic, or any other races and ethnicities not specified), marital status, education (high school or less, or above high school), family income as percentage of federal poverty level (<100%, 100%-124%, 125%-199%, 200%-399%, or ≥400%), insurance coverage (public only, or uninsured or any private), and US Census region (Northeast, Midwest, South, or West), and survey years (2003-2021) were included as covariates but omitted because of limited space. The total sample size included 79 998 individuals aged 65 years and older, equivalent to the population size of 44 069 087, and 2877 individuals with ADRD, equivalent to the population size of 1 516 102, from the Medical Expenditure Panel Survey (MEPS). Total drug expenditures include direct payments for prescription drugs, including OOP payments. ADRD was identified by *International Classification of Diseases, Ninth Revision (ICD-9)* codes 290, 294, 331, and 797 and *International Statistical Classification of Diseases and Related Health Problems, Tenth Revision (ICD-10)* codes F01, F03, G30, and G31. Depression was identified by *ICD-9* codes 296, 300, 309, and 311 and *ICD-10* codes F32, F33, F34, F41, and F43. Diabetes, hypertension, and heart disease (coronary heart disease, angina, myocardial infarction, stroke, and other unspecified heart disease) were identified by the priority condition variables in MEPS.

## Discussion

This population-based cross-sectional study found that ADRD was associated with significantly increased total and OOP drug expenditures, with varying levels of financial burden across cost distributions. The incremental drug costs associated with ADRD were disproportionately higher at the upper end of the cost distribution. Coexisting conditions further increased drug costs among individuals with ADRD along the drug cost distribution,^6^ most notably for diabetes.

Our study has several limitations, including underrepresentation of the population with ADRD in the MEPS data. Owing to the limited sample size, prescription medications were not further categorized. Also, it is important to note that we examined the overall drug expenditures. Future studies may focus on specific drug categories. Furthermore, our analyses could not account for the severity or timing of ADRD diagnosis because of limited data. Policymakers should address the disproportionate prescription burden on patients with ADRD, which may lead to low medication adherence and worsening health.^2,6^ Targeted policies are needed to alleviate financial strain on patients, families, and Medicare, especially for those with coexisting conditions.^6^

## Notes

Supplement.
Data Sharing Statement
