# Supplementary material for: Prescription Drug Costs Among People With Alzheimer Disease and Related Dementias
Source: JAMA Netw Open. 2024 Sep 12;7(9):e2433026. doi: 10.1001/jamanetworkopen.2024.33026 (PMC11393716; doi:10.1001/jamanetworkopen.2024.33026)
Supplement: Supplement. — Data Sharing Statement [file jamanetwopen-e2433026-s001.pdf]

Data Sharing Statement

Jang. Prescription Drug Costs Among People With Alzheimer Disease and Related Dementias. *JAMA Netw Open*. Published September 12, 2024. doi:10.1001/jamanetworkopen.2024.33026

Data
Data available: No

Additional Information
Explanation for why data not available: The data used in our study is publicly available at the Agency for Healthcare Research and Quality (AHRQ)'s Medical Expenditure Panel Survey (MEPS) website.
